# Distribution and diversity of enzymes for polysaccharide degradation in fungi

**DOI:** 10.1038/s41598-017-00258-w

**Published:** 2017-03-16

**Authors:** Renaud Berlemont

**Affiliations:** Department of Biological Sciences, California State University, Long Beach, Long Beach, USA

## Abstract

Fungi are important polysaccharide degraders in the environment and for biotechnology. Here, the increasing number of sequenced fungal genomes allowed for systematic identification of genes and proteins involved in polysaccharide degradation in 218 fungi. Globally, 9,003 sequences for glycoside hydrolases and lytic polysaccharide mono-oxygenases targeting cellulose, xylan, and chitin, were identified. Although abundant in most lineages, the distribution of these enzymes is variable even between organisms from the same genus. However, most fungi are generalists possessing several enzymes for polysaccharide deconstruction. Most identified enzymes were small proteins with simple domain organization or eventually consisted of one catalytic domain associated with a non-catalytic accessory domain. Thus unlike bacteria, fungi's ability to degrade polysaccharides relies on apparent redundancy in functional traits and the high frequency of lytic polysaccharide mono-oxygenases, as well as other physiological adaptation such as hyphal growth. Globally, this study provides a comprehensive framework to further identify enzymes for polysaccharide deconstruction in fungal genomes and will help identify new strains and enzymes with potential for biotechnological application.

## Introduction

Glycoside hydrolases (GHs) and lytic polysaccharide mono-oxygenases (LPMOs) with other carbohydrate active enzymes (e.g., polysaccharide lyases), are essential for the processing of polysaccharides^1^. Among the many identified polysaccharides, cellulose and xylan from plants represent the major source of carbon in land ecosystems. Chitin, produced by arthropods and fungi, is an important source of carbon and nitrogen in both marine and land ecosystems. The enzymatic degradation of these polysaccharides is essential for many ecosystem-processes including nutrient cycling (e.g., carbon cycling)^2^ and herbivores nutrition^3^.

In order to degrade polysaccharides, many enzymes with synergistic action are required. For example GHs with an endo-mode of action (e.g., endo-cellulase) and GHs active on extremities (e.g., exo-cellulase) act synergistically to release short oligosaccharides. Finally some GHs are involved in the processing of these shorter degradation products (e.g., β-glucosidase). In consequence, most identified polysaccharide degraders are equipped with several GH families^1, 4, 5^. Often, polysaccharides associate and form complex super-structures (e.g., cellulose and xylan in plant cell walls); the deconstruction of these complex structure requires further synergy between enzymes targeting chemically distinct but physically associated substrates. Hence, many degraders often target several substrates (e.g., cellulose and xylan)^5, 6^.

In the environment, the hydrolysis of cellulose, xylan, and chitin is mostly supported by bacteria and fungi. Several strategies have been described: the production of (i) individual enzymes, sometimes associated with non-catalytic accessory domains (i.e., multidomain GHs)^7, 8^, (ii) the production of multiactivity GHs with several catalytic domains associated; and (iii) the synthesis of non-covalent multi-protein complexes called cellulosomes^9, 10^. Multidomain/activity GHs, and cellulosomes are promising tools for improving the deconstruction of biopolymers and biofuel industries^9, 11–13^.

Beside GHs, CAZymes include some proteins with “auxiliary activities” (AAs), among others. The proteins are involved in lignin deconstruction and oxidative degradation of cellulose and chitin (i.e., lytic polysaccharide mono-oxygenases, LPMOs)^1^. Proteins from AA family 9 and 10 are LPMOs, previously classified as GH family 61 and CBM33, respectively. According to CAZy DB^1^, AA family 9 is exclusively observed in eukaryote whereas AA family 10 is found mostly in bacteria. Finally, AA13 is the third family of enzyme with LPMO activity and contains only a few identified sequences.

The biochemical characterization of many proteins from several GH and AA families and the identification of homologous sequences allowed the creation of HMM profiles for GH and AA families. These HMM profiles can be used to identify sequences with specific GH and AA domains^14, 15^. In addition, many GH families display substrate specificity. Thus, the potential activity of a protein can be determined by identifying its GH and/or LPMO domains. More precisely, according to the CAZy DB, most characterized proteins from GH families 5, 6, 7, 8, 12, 44, 45, and 48 are cellulases. Next, GH families 10, 11, and 30 are xylanases, whereas GH families 18, 19, and 85 are mostly chitinases^1, 5^. Finally, all biochemically characterized AA9s are active on cellulose whereas AA10s are either cellulases or chitinases^1^.

Recently, the systematic analysis of sequenced bacterial genomes highlighted the distribution and the variability of GHs involved in cellulose, xylan, and chitin degradation^5, 6, 10^. This approach provides a comprehensive framework to identify the functional potential of sequenced bacteria, to investigate the variation in multidomain and multiactivity GHs, and to identify new enzymes with potential for industrial deconstruction of biopolymers. However, fungi are also essential drivers of the polysaccharide deconstruction in environment^16, 17^ and thus many strains with high hydrolytic activities have been isolated and characterized for biotechnological applications^18^. In this context, the recent increase of sequenced fungal genomes^19^; the development of robust gene-identification algorithms [e.g. ref. 20], and consistent annotation platform [e.g. ref. 21] provide an unprecedented opportunity to investigate both the distribution of enzymes involved in carbohydrate deconstruction and their domain organization in fungi. In July 2016, 218 sequenced fungal genomes were publically accessible, and hundred were being processed, on the MycoCosm portal^19^. These strains were sequenced in order to (i) better understand the plant-fungi interaction (e.g., phytopathogens and mycorhizal symbionts), (ii) provide new insight into the conversion of biopolymer (e.g., plant cell wall biorefinery), and (iii) mine the potential of the yet undiscovered natural arsenal of potential application (e.g., antibiotic)^19^. Globally, as stated on the MycoCosm portal, “these sequenced genomes represent a rich source of valuable metabolic pathways and enzyme activities that will remain undiscovered and unexploited until a systematic survey of phylogenetically diverse genome sequences is undertaken”. Here, the procedure developed by Talamantes *et al.* for identification of glycoside hydrolases in sequenced bacterial genomes was applied in order to identify potential enzymes for cellulose, xylan, and chitin deconstruction in sequenced publically accessible fungal genomes^10, 19^.

First the distribution of potential enzymes across genomes was investigated. Chitinases, involved in both chitin degradation and fungal cell-wall metabolism, were hypothesized to be abundant in most lineages. The distribution of other traits was expected to reflect niche adaptation, as described in bacteria^6^. Next, the taxonomic conservatism of sequences involved in polysaccharide deconstruction across taxa was investigated. Closely related strains were expected to share similar traits. Finally, we investigated the association of domains in GHs and LPMOs. As for many bacterial polysaccharide degraders^10^, fungi were expected to display abundant and diverse sets of proteins and proteins architectures including many multi-domain and multi-activity enzymes.

## Results

### Enzymes identification

In 218 fully sequenced fungal genomes, 3,607, 1,060, and 2,386 domains for GH targeting cellulose, xylan, and chitin were identified. In addition, 1,974 lytic polysaccharide mono-oxygenases (i.e., LPMO) were detected. These 9,027 identified catalytic domains were associated with several other catalytic domains and many non-catalytic domains (e.g., carbohydrate binding modules - CBMs) and corresponded to 9,003 proteins (see Table 1, Supplementary data). Most domains targeting cellulose belonged to GH family 5. Domains from GH families 7, 12, 45, and 6 were intermediate whereas fewer potential cellulases from GH families 8, 9, 44, and 48 were identified. Potential xylanases were mostly from GH family 10. However, many GH11s and GH30s were also detected. Finally, most potential chitinases were from GH family 18, with reduced number of enzymes from GH85, and no detected domain from GH family 19. Finally, most identified LPMOs were AA9s (targeting cellulose) and few were AA10s (targeting cellulose or chitin).

**Table 1:** Identification of domain for cellulose, xylan, and chitin deconstruction in sequenced fungal genomes.

Domain (PFamID) #dom./#prot. % of 1-Domain	Multi-activity enzymes (Domain architecture)	#prot.	Example (Strain – Gene id)
GH5 (PF00150) 2263/2255 85%	CBM1–2(GH5)	2	*Sistotremastrum niveocremeum* HHB9708 ss-1_1.0 (Sisni1|484442)
	CBM1-GH5-GH6	2	*Sistotremastrum suecicum* (Sissu1|1057361)
	2(GH5)	4	*Rhizophagus irregularis* DAOM 181602 (Gloin1|64734)
	2(GH5)−2(CBM10)	2	*Orpinomyces sp.* (Orpsp1_1|1180573)
GH6 (PF01341) 249/249 54%	GH3-CBM10-GH6–2(CBM10)	1	*Orpinomyces sp.* (Orpsp1_1|1176522)
	CBM1-GH5-GH6	2	*Sistotremastrum niveocremeum* HHB9708 ss-1 1.0 (Sisni1|485627)
GH7 (PF00840) 439/439 69%	GH13-CBM20-GH7	1	*Phaeomoniella chlamydospora* UCRPC4 (Phach1|4371)
GH8 (PF01270) 12/12 100%			
GH9 (PF00759) 82/82 87%			
GH10 (PF00331) 531/527 67%	CBM1–2(GH10)	2	*Sebacina vermifera* MAFF 305830 (Sebve1|110734)
	CBM1–3(GH10)	1	*Piriformospora indica* DSM 11827 from MPI (Pirin1|75864)
	GH11-GH10–2(CBM10)	1	*Orpinomyces sp.* (Orpsp1_1|1191833)
GH11 (PF00457) 382/373 82%	GH11–3(CBM10)-GH11	2	*Orpinomyces sp.* (Orpsp1_1|1184756)
	GH11–2(CBM10)-GH11	1	*Orpinomyces sp.* (Orpsp1_1|1175252)
	GH11-CBM10-GH11-CBM10-GH11	1	*Orpinomyces sp.* (Orpsp1_1|1175428)
	GH11-GH10–2(CBM10)	1	*Orpinomyces sp.* (Orpsp1_1|1191833)
	2(GH11)	4	*Allomyces macrogynus* ATCC 38327 (Allma1|15884)
GH12 (PF01670) 376/376 96%			
GH18 (PF00704) 2290/2284 75%	2(GH18)	6	*Puccinia striiformis f. sp. tritici* PST-130 (Pucst1|501894)
	GH18-GH25	5	*Chaetomium globosum* v1.0 (Chagl_1|16288)
	GH18-GH81	1	*Macrophomina phaseolina* MS6 (Macph1|905)
	GH25-CBM18-GH18	9	*Chaetomium globosum* v1.0 (Chagl_1|12853)
	GH25-GH18	22	*Trichophyton verrucosum* HKI 0517 (Triver1|3242)
	2(GH25)-CBM18-GH18	2	*Volvariella volvacea* V23 (Volvo1|121331)
	2(GH25)-GH18	1	*Eutypa lata* UCREL1 (Eutla1|3713)
	8(GH25)-GH18	1	*Allomyces macrogynus* ATCC 38327 (Allma1|9612)
GH30 (PF02055) 147/147 75%			
GH44 (PF12891) 26/26 85%			
GH45 (PF02015) 148/148 71%			
GH48 (PF02011) 11/11 54%			
GH85 (PF03644) 96/96 97%			
AA9 (PF03067).1803/1795 79%	AA9-AA9	7	*Melampsora lini* CH5 (Melli1|198847)
AA10 (PF03443) 171 /171 79%			

Globally fungi represent a rich reservoir of GHs and LPMOs for cellulose, xylan, and chitin deconstruction dominated by GH family 5, 10, 18, and AA family 9 respectively. In addition, the number of identified domains deviated from the number of identified proteins suggesting that some proteins contains several catalytic domains (i.e., multi-activity) and in some case some accessory non-catalytic domains (e.g., CBM).

This suggests that both fluctuation in the genome content (i.e., the number of catalytic domain per genome) and the enzymes multi-domain architecture (i.e., the association of catalytic domains with other domains) could affect the fungal potential for polysaccharide deconstruction.

## Enzymes distribution

As of June 2016, the set of publically accessible genomes retrieved from the MycoCosm portal contained 218 genomes (Supplementary data). This collection of genomes was biased towards 2 major phyla: (i) the phylum Ascomycota (n = 120 genomes) containing the subphyla *Pezizomycotina* (n = 102 genomes), *Saccharomycotina* (n = 13), and *Taphrinomycotina* (n = 5) and the phylum (ii) Basidiomycota (n = 85) containing *Agaricomycotina* (n = 69), *Pucciniomycotina* (n = 9), and *Ustilaginomycotina* (n = 6). A few genomes from deeply branched clades including *Mucoromycotina* (n = 6) and *Kickxellomycotina* (n = 1) and some genomes from the phyla Blastocladiomycota, Entomophthoromycota, Chytridiomycota, Neocallimastigomycota, Glomeromycota, Cryptomycota were also analyzed.

**Figure 1:**
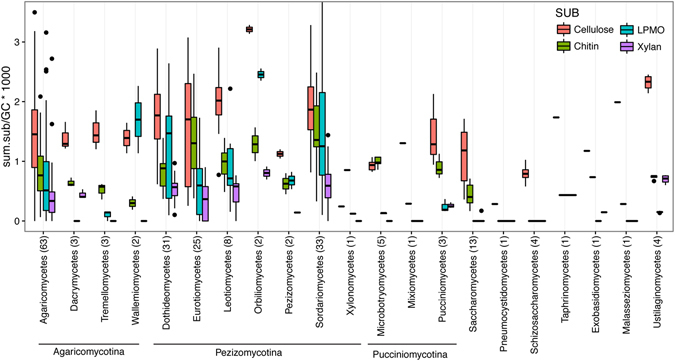
Frequency of GH domains (per 1,000 predicted genes) involved in cellulose, xylan, and chitin deconstruction and LPMO domains in fungal genomes from major classes (numbers in parentheses stand for the number of sequenced genomes).

We first investigated distribution of the functional potential in sequenced fungal genomes. Cellulases, accounting for ~0.15% of genes in analyzed fungi (median value, Fig. 1), were the most frequent identified traits. However genomes from the class *Orbiliomycetes* (n = 2) and *Ustilaginomycetes* (n = 4) displayed higher cellulase frequency. Chitinases, identified in all genomes except in members of the classes *Malasseziomecetes* (n = 1) and Schizosaccharomycetes (n = 4), accounted for ~0.1% of the genes in analyzed genomes. The frequency of LPMOs was variable. For example, in the subphylum *Agaricomycotina*, members of the *Dacrymycetes* (n = 3), and *Tremellomycetes* (n = 3) displayed reduced number of LPMO, Wallemiomycetes (n = 2) displayed high frequency whereas the frequency of LPMO in *Agaricomycetes* (n = 61) was intermediate. Finally, the frequency of xylanase was reduced in most genomes.

Despite variations, the number of identified domains for cellulose, xylan, and chitin deconstruction correlated with the genomes size (expressed as the total number of predicted genes, Table 2, Figure S1): larger genomes had more cellulases, xylanases, chitinases, and LPMOs than small genomes. In addition, the frequency of traits correlated with each other (Table 2). However, across subphyla distinct trends were observed. For example, in members of the subphylum *Pezizomycotina* (n = 102 genomes, ~8,000 to ~15,000 genes/genome) the frequency of identified domains significantly correlated with the number of predicted genes (*r*_*s*_ from 0.28 for chitinases to 0.58 for cellulases). In addition, the frequency of cellulases, xylanases, and LPMOs were highly correlated with each other (*r*_*s*_ from 0.78_Cellulase:Xylanase_ to 0.8_Xylanase:LPMO_, all significant). However, although significant, the frequency of chitinases was less correlated with the other traits (*r*_*s*_ from 0.29_Chitinase:LPMO_ to 0.40_Cellulase:Chitinase_). In the subphylum *Agaricomycotina* (n = 69 genomes, ~5,000 to ~35,000 genes/genomes) the number of identified domains and the number of predicted genes were not correlated. Nevertheless, the frequency of cellulases, xylanases, and LPMOs were highly correlated with each other (*r*_*s*_ from 0.65_Xylanase:LPMO_ to 0.8_Cellulase:Xylanase_, all significant). The correlations between chitinases and other functional traits of interest were reduced (*r*_*s*_ ranging from 0.4_Chitinase:LPMO_ to 0.52_Chitinase:Cellulase_).

**Table 2:** Correlation between traits and traits vs. number of predicted genes count (GC) (all significant, p < 0.05).

Spearman/Pearson	Cellulase	Xylanase	Chitinase	LPMO	GC
Cellulase		0.84/0.81	0.59/0.47	0.78/0.60	0.48/0.44
Xylanase			0.55/0.26	0.79//0.49	0.39/0.27
Chitinase				0.52/0.42	0.42/0.39
LPMO					0.36/0.22

**Figure 2:**
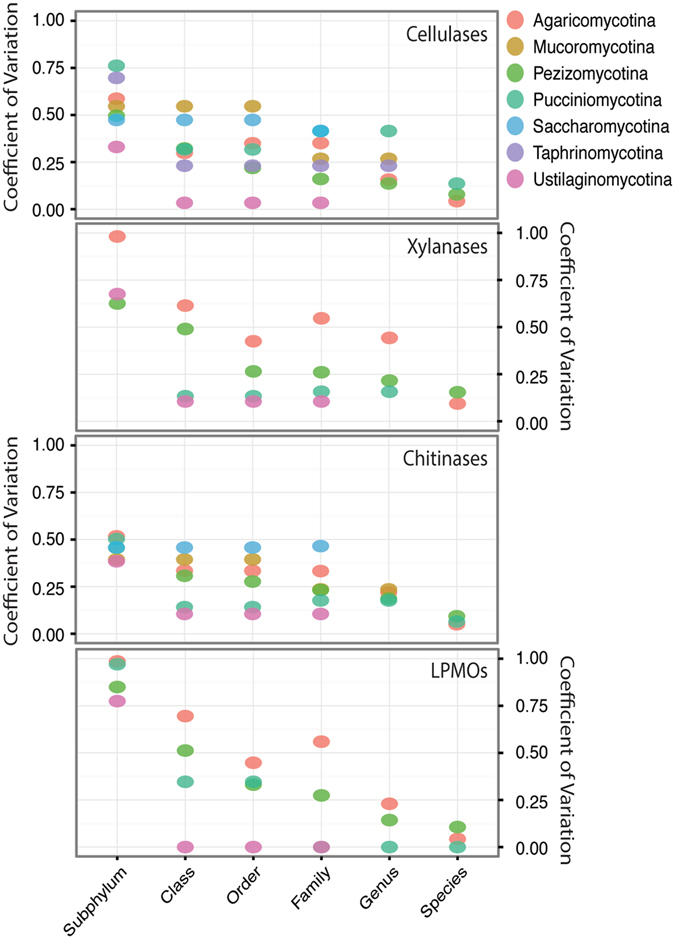
Conservatism of enzymes for cellulose, xylan, and chitin deconstruction in in taxa with more than 1 sequenced genomes, from subphylum to species. The coefficient of variation is the taxon specific standard deviation divided by the taxon specific mean value.

Next, the conservatism polysaccharide deconstruction potential, based on predicted cellulases, xylanases, chitiniases, and LPMOs, across taxonomic ranks was investigated. Taxa with more than one sequenced genome (per taxon), from subphylum to species, were analyzed (Fig. 2). At low taxonomic resolution (e.g., subphylum, class) the genomes specific distribution of cellulases, xylanases, chitinases, and LPMOs was highly variable except in taxa with few strains, for example in the subphylum *Ustillaginomycotina* (n = 6 genomes), with 4 genomes were from the family *Ustilaginaceae*. Conversely, increasing the taxonomic resolution reduced the variation among genomes (Fig. 2). GHs for cellulose and chitin were the most conserved traits, whereas GHs for xylan and LPMOs displayed high CoV in many groups. In several taxa, the reduced number of sequenced genomes [e.g., Ustillagomycotina (n = 6 genomes), Taphrinomycotina (n = 5)], limited the comparison. However, among Pezizomycotina (n = 102) traits were conserved (CoV < 0.25) within families whereas in Agaricomycotina (n = 69), traits were mostly conserved at the genus level. Thus, in most characterized lineages, identifying the high-rank taxonomic affiliation of fungi does not allow for accurate estimation of the potential for carbohydrate utilization. In most cases, conserved trait distribution is observed at the genus or species level.

The correlation between genome size and the number of identified cellulases, xylanases and LPMOs suggests that polysaccharide deconstruction is an essential, conserved, function in most fungi. Finally, potential for chitin processing deviates from the other potentials thus highlighting the role of chitin as both an important component of the cell wall and a nutrient. Strains associated with the potential to target one type of polysaccharide have the potential to target all the substrates. In addition, these strains are associated with apparently redundant enzymes targeting the same substrate. However, few lineages have reduced potential to target the identified substrates in all major lineages. Beside strains with reduced potential for polysaccharide deconstruction, variation in the frequency of traits of interest suggested that strains from the same class have evolved varying strategies for carbohydrate processing.

**Figure 3:**
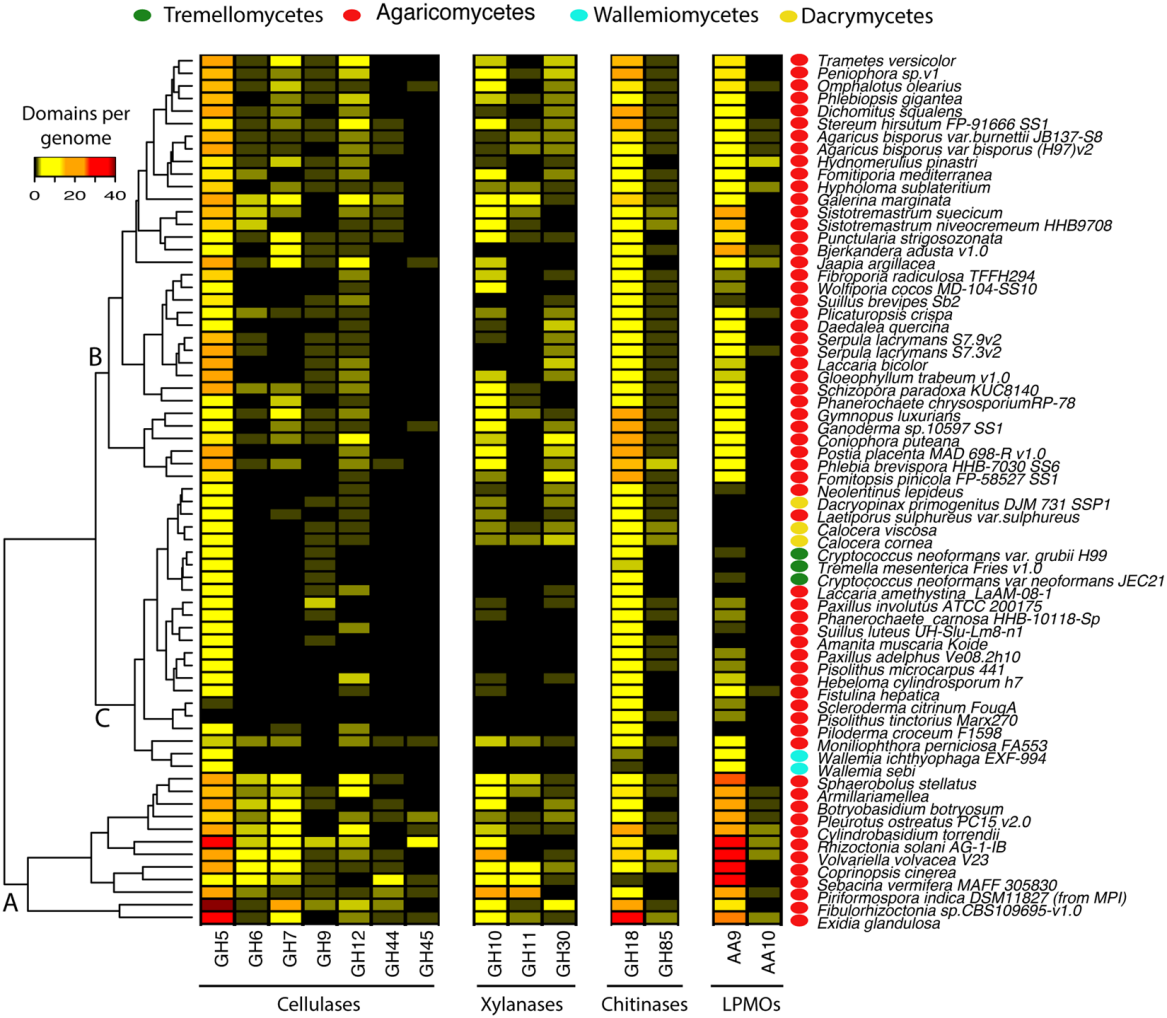
Distribution of domains involved in the deconstruction of cellulose, xylan, and chitin, in sequenced genomes from the subphylum Agaricomycotina.

Next, the genome-specific distribution of traits for polysaccharide processing was investigated. The clustering of strains from the subphylum *Agaricomycotina* (n = 69 genomes) highlighted three major clusters (Fig. 3). The first group (A) was composed of 12 *Agaricomycetes* strains including, *Volvariella volvacea* V23 and *Exidia glandulosa*, and displayed high numbers of cellulases (mostly GH5, 6, and 7), xylanases (mostly GH10 and some GH11), chitinases (mostly GH18), and LPMOs (mostly AA9). These strains also contained several GH44s, GH45s, and AA10s. The second group (B, n = 34 genomes), including *Trametes versicolor* and *Agaricus bisporus*, displayed intermediate diversity and frequency of identified traits, with GH5s, GH18s, and LPMO61s dominating. Although reduced the other GH and LPMO domains were still detected. Finally, the remaining genomes (cluster C), including *Tremella mesenterica* and members of the *Calocera* genus, displayed high frequency of GH5, GH18, and few AA9 but further reduced frequency of the other traits. Members of the Agaricomycetes were detected in all three clusters whereas the few other classes (e.g., *Dacrymycetes*) were mostly found in the cluster associated with reduced frequency and diversity of traits for carbohydrate utilization (i.e., cluster C).

**Figure 4:**
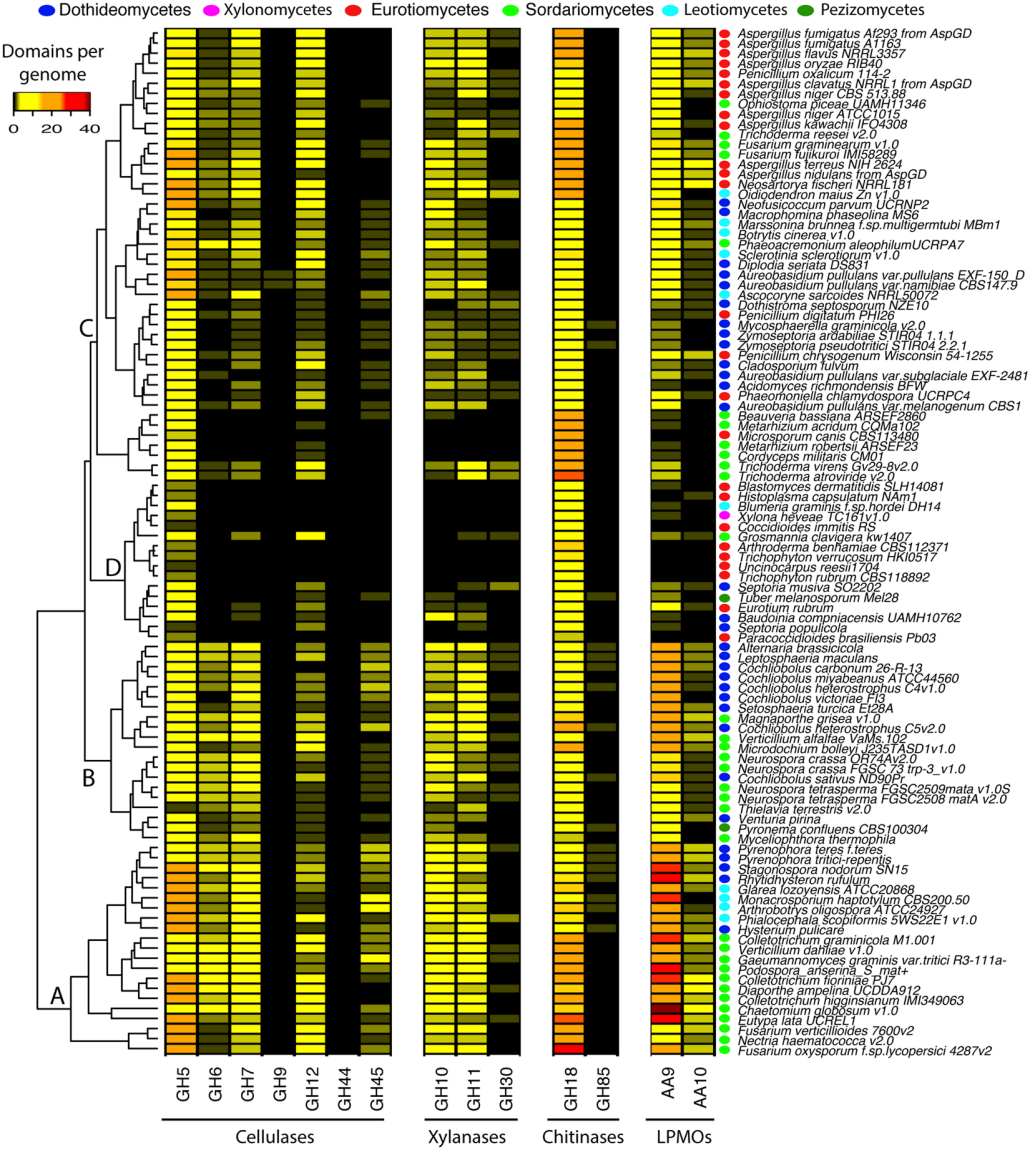
Distribution of domains involved in the deconstruction of cellulose, xylan, and chitin, in sequenced genomes from the subphylum Pezizomycotina.

Regarding genomes in the subphylum *Pezizomycotina* (Fig. 4), the analysis revealed a slightly different clustering with 4 main groups. The first cluster (A, n = 21 genomes), containing *Chaetomium globosum* and *Fusarium oxysporum*, displayed high frequency and diversity of both GH and LPMO domains. Then, 2 intermediate clusters were identified. In the first intermediate cluster (B), containing *Alternaria brassicicola and several Cochliobolus*, LPMO were the most abundant enzymes. In the second intermediate cluster (C), including several *Aspergillus*, genomes were dominated by GH domains and yet contained several LPMO domains. Finally, the last cluster (D), including *Xylona* (class *Xylonomycetes*) and *Blumeria* (class *Leotomycetes*) displayed genomes with reduced numbers of predicted enzymes except potential few cellulases from GH family 5 and chitinases from GH family 18.

In other strains (Figure S2), most genomes displayed reduced frequency and diversity of GHs for cellulose, xylan, chitin, and LPMO. Cellulases from GH family 5 and chitinases from GH family 18 were to most abundant identified sequences.

## Enzyme multi-domain architecture

Finally, we systematically investigated the domain association in fungal cellulases, xylanases, chitinases, and LPMOs. Among the 9,003 identified proteins, 1,936 were multi-domain proteins with at least two domains identified. Between ~20 and ~45% of the GHs from families 5, 6, 7, 10, 18, 45, 85, and LPMOs were identified in multi-domain proteins whereas more than 80% of the domains from GH families 8, 9, 11, 12, 30, and 44 were single domain proteins (Tables 1, S1, Supplementary data). Most multi-domain enzymes consisted of a single catalytic domain associated with non-catalytic accessory domain(s). More precisely, 1,425 proteins contained at least one CBM domain including 1,123 proteins with at least one CBM1 domain targeting cellulose. This CBM was observed in association with all the types of catalytic domain of interest, except with domains from GH families 8, 9, 48, and 85. Next, 78 proteins were associated with CBM10, also targeting cellulose. CBMs from families 18, 19, and 5/12, targeting chitin, were found associated with ~17% of identified potential chitinases from GH family 18. Other CBMs from families 20, 4/9, and X2 were also detected but at a lower frequency. Among others, 32 proteins with potential to target cellulose (i.e., cellulases or AA9s) were associated with CBM from family 20 and several potential cellulases from GH family 5 were associated with CBM X2.

Among the 203 multi-domain architecture types identified (Table S1, Supplementary data), 126 were observed only once including one AA9 associated with 28 CBM1s identified in *Arthrobotrys oligospora* ATCC 24927 (Artol1|5689).

Reduced numbers of multi-activity proteins with several catalytic domains, sometime associated with accessory non-catalytic domains were identified. Multi-activity proteins were mostly assemblies of similar domain (e.g., GH5-GH5, AA9-AA9), and few were hetero-GHs (i.e., several distinct GH domains). One GH5/GH6 potential hetero-cellulase was identified in each *Sistotremastrum* (n = 2 genomes) and a GH10/GH11 potential hetero-xylanase in *Orpinomyces sp.*

Beside the domains of interest, 40 potential hetero-chitinases from GH family 18 were associated with domains from GH family 25 (i.e., lysozyme). Other hetero GHs included a potential cellulase_GH7_/amylase_GH13_ in *Phaeominiella chlamydospora*, a potential cellulase_GH6_/β-glucosidase_GH3_ in *Orpinomyces sp.*, and a potential chitinase_GH18_/endo-β−1,3-glucanase_GH81_. Finally, several proteins domains targeting cellulose, xylan, and chitin were associated with other unexpected catalytic domains (Table S1, Supplementary data). For example, a potential cellulase_GH12_/Cu-oxidase in *Puccinia graminis* (Pucgr2|14446), a potential LPMO_AA9_/endo-α−1,4-polygalactosaminidase_GH114_ in *Hysterium pullicare* (Hyspu1_1|113907), a potential cellulase_GH5_/metallo-B-lactamase in *Diplodia seriata* (Dipse1|6209) and a potential cellulase_GH9_/transposase in *Lichtheimia corymbifera* (Liccor1|6997) were identified.

**Figure 5:**
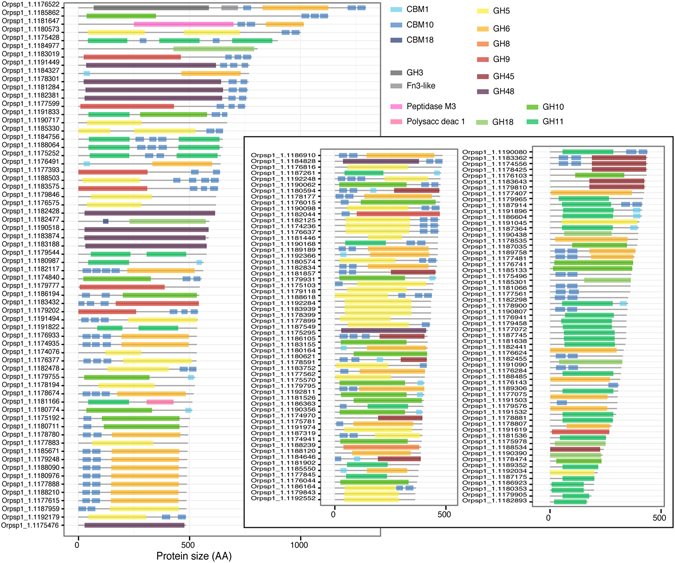
Identification of proteins involved in cellulose, xylan, and chitin deconstruction in *Orpinomyces sp.* (phylum Neocallimastigomycota).

Surprisingly, 38 multi-domain architecture types were identified just in the genome of *Orpinomyces sp.* (phylum Neocallimastigomycota) (Fig. 5). This genome contained the higher number (n = 176) of identified proteins for cellulose, xylan, and chitin processing in this study. When compared to other identified genomes associated with high potential for polysaccharide deconstruction or industrially relevant fungi (Table 3, Figures S3–S11), *Orpinomyces sp.* displayed extremely high frequency of proteins for cellulose, xylan, and chitin deconstruction. In comparison, most fungi, including the industrially important species displayed mostly simple enzymes composed of one unique catalytic domain sometime associated with one CBM (Table 3, Figures S3–S11).

**Table 3:** Identification of proteins for cellulose, xylan, and chitin deconstruction in genomes associated with high potential for polysaccharide deconstruction or industrially relevant fungi.

Strain	Phylum	# proteins- % multidomain prot.	Figure
*Orpinomyces sp.*	Neocallimastigomycota	176–56%	5
*Fibulorhizoctonia sp.* CBS109695	Basidiomycota	113–22%	S3
*Exidia glandulosa*	Basidiomycota	113–27%	S4
*Rhizoctonia solani* AG-1 IB	Basidiomycota	111–28%	S5
*Volvariella volvacea* V23	Basidiomycota	101–42%	S6
*Chaetomium globosum* v1.0	Basidiomycota	100–26%	S7
*Aspergillus oryzae* RIB40	Ascomycota	53–17%	S8
*Postia placenta* MAD698-R v1.0	Basidiomycota	51–10%	S9
*Trichoderma reesei* v2.0	Ascomycota	42–28%	S10
*Myceliophthora_thermophila (Sporotrichum_thermophile*) v2.0	Ascomycota	36–28%	S11

Finally, accounting for multi-domain architecture in GHs and LPMOs for the clustering of sequenced fungal genomes, showed a correlation with the clustering based on the distribution of catalytic domain only (r_mantel_ = 0.87, p < 0.01).

## Discussion

As depicted here, the identification of protein domains based on Hidden Markov Profiles^14^ provides an easy way to predict the activity of sequenced fungi and to investigate the diversity of enzymes for polysaccharide utilization^10, 15^. This approach is versatile and can be adjusted to various needs, at will, by including new genomes and new HMM-profiles. However, this technique suffers some limitations. Among others, not all the GH families have assigned HMM-profiles. For example, no HMM profile was derived, yet, from 21 identified cellulases in eukaryotes from GH family 74, or from the 14 identified LPMOs from AA family 13. However this approach, using the entire Pfam database rather than a customized database only, allows for the identification of previously unsought domains (e.g., metallo-B-lactamase) associated with the domain of interests (i.e., GH, AA). In addition, we have a limited understating of the biochemistry of the 9,003 identified proteins as reduced number of enzymes have been characterized^1^. In addition, although most GH and LPMO families identified here display conserved substrate specificity, characterized proteins sometime deviate from their expected substrate specificity. For example, among the 84 biochemically characterized cellulases from GH family 7, listed on the CAZy DB, 3 proteins are associated with cellulase/chitosanase activity, although no reference is explicitly cited^1^.

This depicted approach allows for the rapid identification of potential genes involved in carbohydrate processing. The analyzed fungal genomes contain from 2 to 170 potential enzymes targeting cellulose or xylan, in *Dekkera bruxellensis* CBS2499, along with other strains, and *Orpinomyces sp.*, respectively. *D. bruxellensis* is mostly active on short substrates^22^ whereas *Orprinomyces sp.* is a well known plant biomass degrader isolated from mammal gut^23^. Cellulases, mostly from GH family 5, were detected in all sequenced genomes but in *Pisolithus tinctorius*, a mycorrhyzal symbiont with known reduced plant cell wall degrading enzymes^24^. Xylanases are the most dispensable traits identified in this study with 61 strains having no identified domain from GH family 10, 11, and 30. Domains for chitinases (i.e., GH18, GH85) were identified in 213 genomes and ranged from 0 in *Schizosaccharomyces* genomes and *Rhizophagus irregularis* DAOM181602 to 34 in *Fusarium oxysporum f. sp. lycopersici* 4287. *Schizosaccharomyces* are fission yeasts producing no chitin^25^ and *R. irregularis* is a plant symbiont with limited hydrolytic capabilities^26, 27^ whereas *F. oxysporum* is a well known chitin degrader^28, 29^. LPMOs, mostly AA9, were identified in 171 genomes. The genome of *Chaetomium globosum*, a cosmopolitan plant material degrader^30^, contains 47 of these potential proteins. Thus, not all sequenced fungi are made equal. However, most have, even limited, potential for multiple polysaccharides deconstruction although 28% of the strains apparently lack the potential to target xylan.

Fungi, and bacteria, are essential drivers of carbon cycling across ecosystems where they secrete enzymes that breakdown complex polysaccharides and release short oligosaccharides. For fungi the processing of chitin, requiring chitinases, is a complex, tightly regulated task, as this polysaccharide is also the main component of the cell wall^31–33^. This supports the high frequency and broad distribution of chitinases in sequenced fungal genomes.

Not all the microbes are directly involved in polysaccharide processing; potential polysaccharide degraders are equipped with all the enzymes for complete polysaccharide breakdown (e.g., cellulases and β-glucosidases)^6, 34^ whereas opportunists are equipped with enzymes involved in the last step of polysaccharide deconstruction only (e.g., β-glucosidases). The opportunists rely on degraders, or their host, to release the substrates^6^. Unlike in sequenced bacterial genomes, dominated by pathogens^5, 35^, 72% of sequenced fungi have the potential to target cellulose, xylan, and chitin and the frequencies of these traits correlate, suggesting that most sequenced fungi can be regarded as potential generalists, targeting multiple polysaccharides. Although, cellulases from GH family 7 are fungi specific^1, 16^, the most frequent identified cellulases in fungi are from GH family 5, as in bacteria^6^. Also, several strains lacking GH7 are associated with cellulases from other families. This suggests that focusing on GH family 7 is likely underestimating the contribution of fungi to the environmental pool of cellulolytic activities in the environment. Similar considerations apply to LPMO as 47 strains lack identified “auxiliary activities”.

Most strains with reduced potential for polysaccharide deconstruction were yeasts including members of the classes *Saccharomycetes, Schizosaccharomycetes, Taphrinomycetes*, and mycorrhizal symbionts (e.g., *P. tinctorius*).

Regarding potential polysaccharide degraders, many fungi, including biotechnologically relevant strains (e.g., *T. reesei*) and important environmental isolates (e.g., *P. placenta*), display many enzymes with simple multi-domain architecture (e.g., GH_x_-CBM_y_). The frequency of multi-activity proteins for polysaccharide processing is extremely reduced in fungi, compared to bacterial polysaccharide degraders (e.g., *Calidcellulosiruptor*, *Clostridium, Bacteroides*)^10^. One notable exception is *Orpinomyces sp.* inhabiting the mammal gut and sharing many genes with bacteria from the same ecosystem (e.g., *Clostridium, Ruminococcus*)^23^. Globally, the reduced frequency of multi-domain proteins for polysaccharide deconstruction in most fungi suggests that their ability to degrade polysaccharide likely results from the apparent functional redundancy of their traits and other mechanisms including the high frequency of auxiliary activity (i.e., LPMO)^16^, and their filamentous growth^36^.

Performing the systematic investigation of 218 sequenced fungal genomes provided an unprecedented opportunity to identify the distribution and the diversity of functional traits involved in polysaccharide deconstruction. However, not all the fungal lineages are evenly represented in this study. Indeed for example, 79% of characterized genomes derive from the Agaricomycotina and Pezizomycotina subphyla. Conversely, several taxa are associated with reduced number of sequenced genomes (e.g., 1 genome in the phylum Neocallimastigomycota: *Orpinomyces sp*.). However, many more genomes will be sequenced as part of the “1,000 Fungal Genomes Project” (1000.fungalgenomes.org), and made publicly accessible thru the Mycocosm portal. More precisely, as of October 2016, ~500 additional genomes are being processed on the Mycocosm portal and several are from poorly characterized clades, including 4 genomes related to *Orpinomyces sp.* The characterization of these additional genomes will further improve our understanding of the distribution and the diversity of traits for polysaccharide processing.

## Material and Methods

### GH identification

Proteins sequences, from “filtered best model”, for publicly accessible sequenced fungal genomes were retrieved from the MycoCosm portal^19^. The protein sequences were analyzed using previously described bioinformatic pipeline aimed at identifying proteins involved in cellulose, xylan, and chitin processing^10^. In addition, lytic polysaccharide mono-oxygenases^37^ [LPMO, AA9 (PF03067) and AA10 (PF03443)] were included in the study. Briefly, first proteins sequences from publically accessible fungal genomes were downloaded from the MycoCosm portal^19^. Next, potential proteins associated with domains of interest were identified by performing an HMMscan^15^ against a custom database containing Hidden Markov Profiles for the domains of interest (i.e., GHs and AA), retrieved from the Pfam A database^14^. Then, positive hits were reanalyzed against the entire Pfam A database to confirm the domain annotation and to identify potential accessory domains not listed in the custom database. Finally, identified domains with e-value <=10^−5^ and alignment coverage >50% of Pfam length were used in subsequent analyses. Substrate specificity of identified GH and CBM domains was derived from biochemically characterized bacterial homologs as described in the CAZy DB^1, 5^: GH 5, 6, 7, 8, 9, 12, 44, 45, and 48 were identified as cellulase; GH 10, 11, and 30 were identified as xylanase; and GH 18, 19, and 85 were identified as chitinases. AA9 were cellulases and AA10 were cellulases/chitinases.

Identified sequences of interest mentioned in the article can be retrieved directly from the MycoCosm portal^19^ using the gene IDs (e.g., Orpsp1_1.1182428) used in the text, in the figures, and in Supplementary data on the MycoCosm portal (http://genome.jgi.doe.gov/programs/fungi/index.jsf). Finally, the complete taxonomy of each individual strain was retrieved from the NCBI taxonomy server (http://www.ncbi.nlm.nih.gov/Taxonomy/) using the “taxize“ and “myTAI“ packages for the R statistical program^38^.

### Statistical analysis

GH distribution and domain organization in sequenced bacterial genomes were analyzed using Vegan, Stats, and APE packages in the R software environment^38, 39^. Clustering strains used two distinct approaches. First, genomes were clustered according to the distribution of GH domains per genome, regardless of the protein architecture. Second, we compared the architecture of all identified proteins with GH domains for cellulose, xylan and chitin, including accessory domains, and then clustered the sequenced genomes as described before^10^. In order to investigate correlation among clusterings we performed Mantel correlation tests (999 permutations) on distance matrixes used for clustering.

## Electronic supplementary material

Supplementary Information

Supplementary Information
