# Supplementary material for: Distribution and diversity of enzymes for polysaccharide degradation in fungi
Source: Sci Rep. 2017 Mar 16;7:222. doi: 10.1038/s41598-017-00258-w (PMC5428031; doi:10.1038/s41598-017-00258-w)
Supplement: Supplementary file 1 — Supplementary Information [file 41598_2017_258_MOESM1_ESM.pdf]

Supplementary Information

Distribution and diversity of enzymes for polysaccharide degradation in fungi

Renaud Berlemont[1*]

Department of Biological Sciences, California State University – Long Beach, Long Beach, USA.

Corresponding author:
Renaud Berlemont
Dept. of Biological Sciences
1250 Bellflower Blvd.
California State University,
Long Beach, CA 90840-9502
562-985-7093
Renaud.berlemont@csulb.edu

Figure S1. Genome-specific frequency (/10,000 predicted genes) of identified protein with domain involved in cellulose, xylan, and chitin deconstruction.

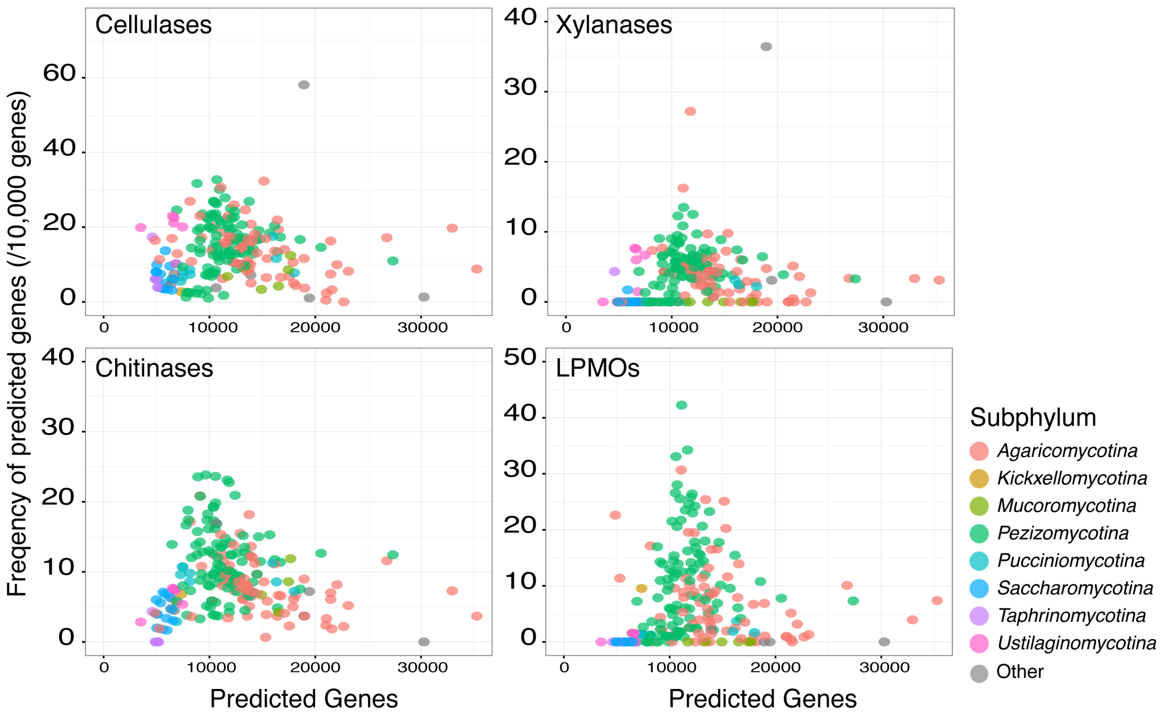

Figure S2. Distribution of domains involved in the deconstruction of cellulose, xylan, and chitin, in sequenced fungal genomes.

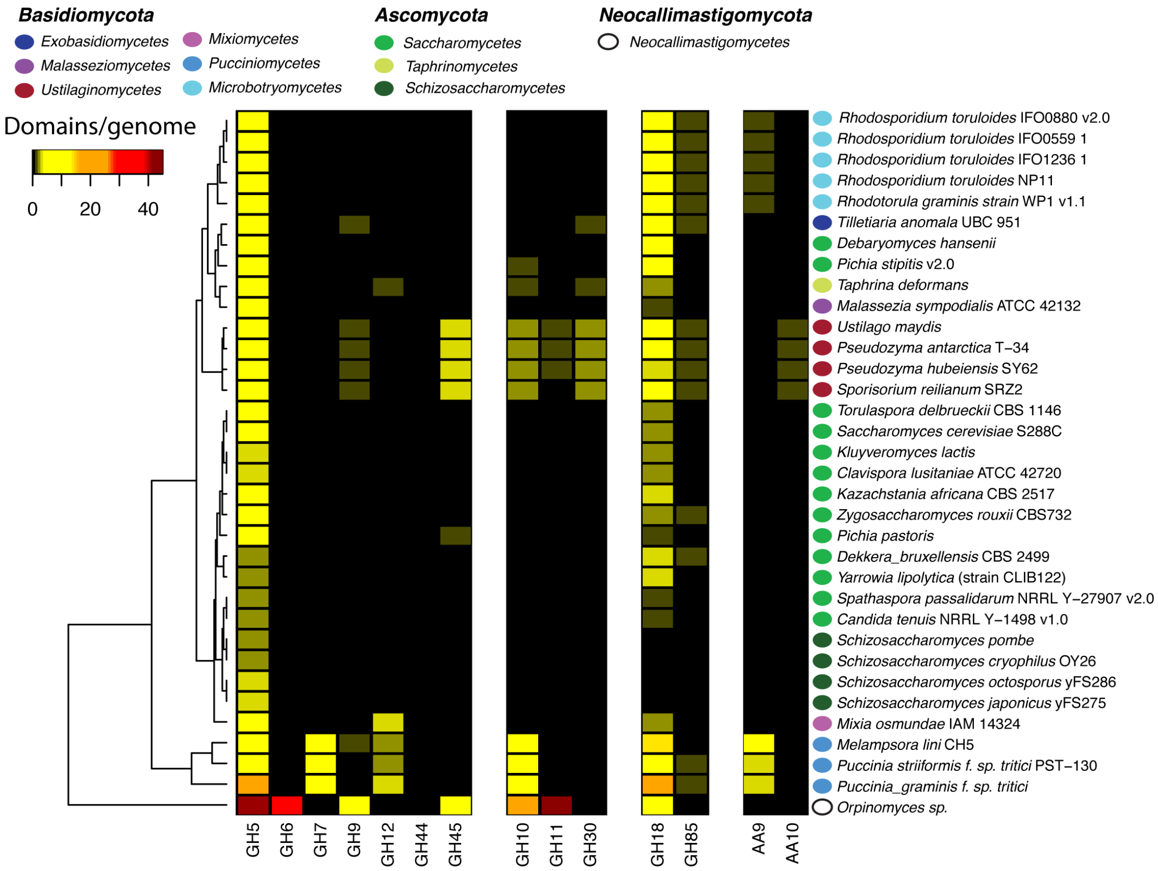

Figure S3. Identification of proteins involved in cellulose, xylan, and chitin deconstruction in *Fibulorhizoctonia sp.* CBS109695 (phylum Neocallimastigomycota).

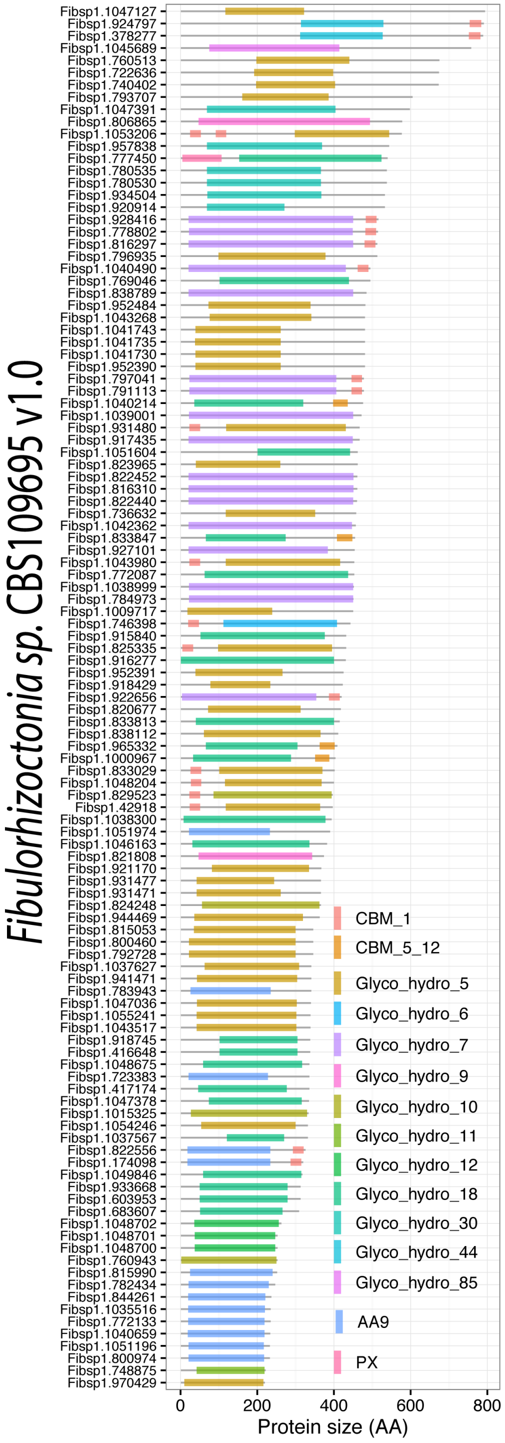

Figure S4. Identification of proteins involved in cellulose, xylan, and chitin deconstruction in *Exidia glandulosa* (phylum Basidiomycota).

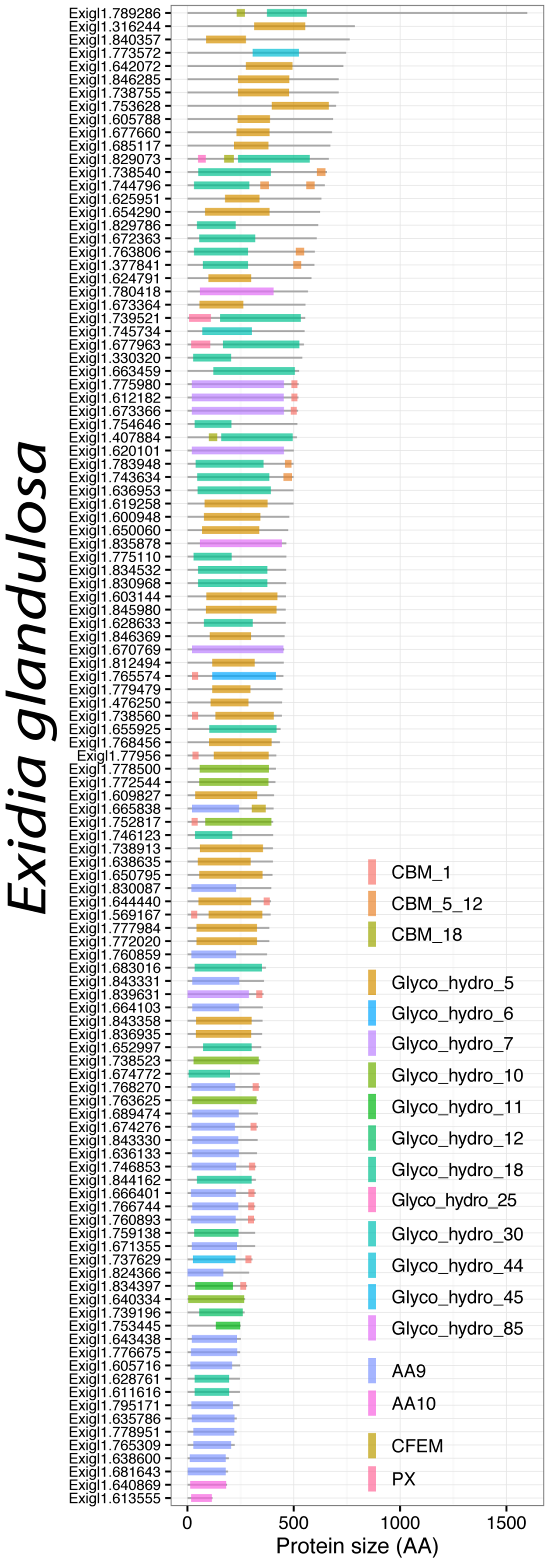

Figure S5. Identification of proteins involved in cellulose, xylan, and chitin deconstruction in *Rhizoctonia solani* AG-1 IB (phylum Basidiomycota).

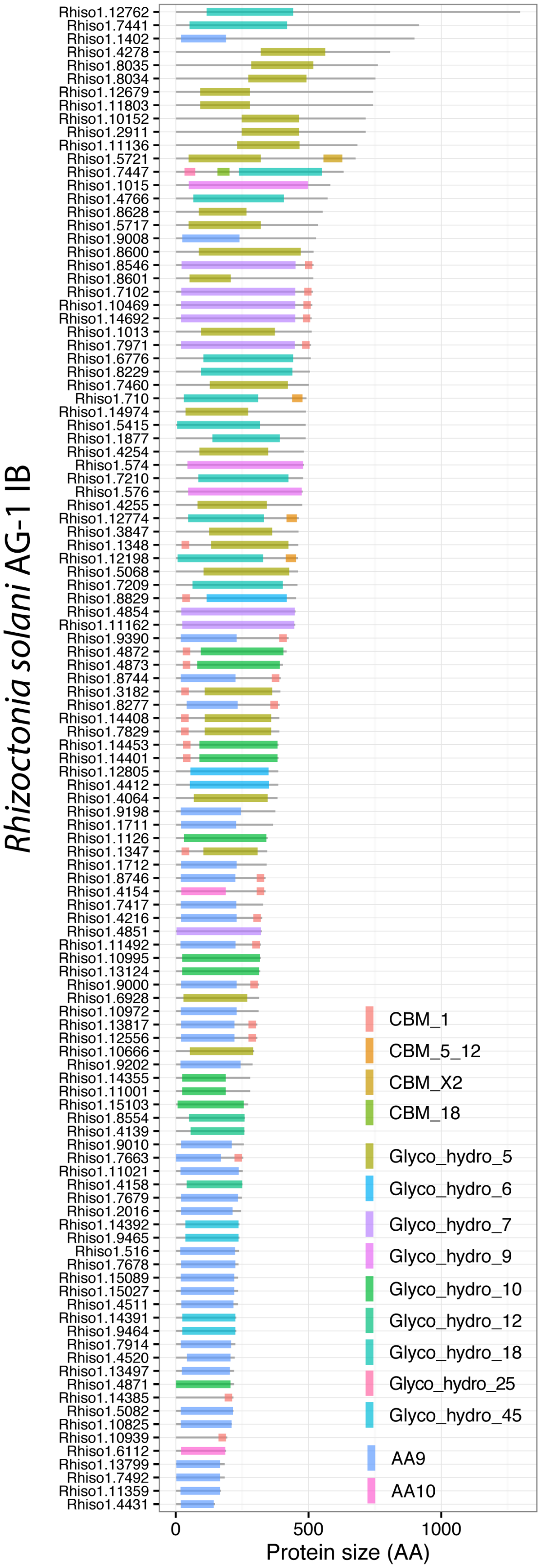

Figure S6. Identification of proteins involved in cellulose, xylan, and chitin deconstruction in *Volvariella volvacea* V23 (phylum Basidiomycota).

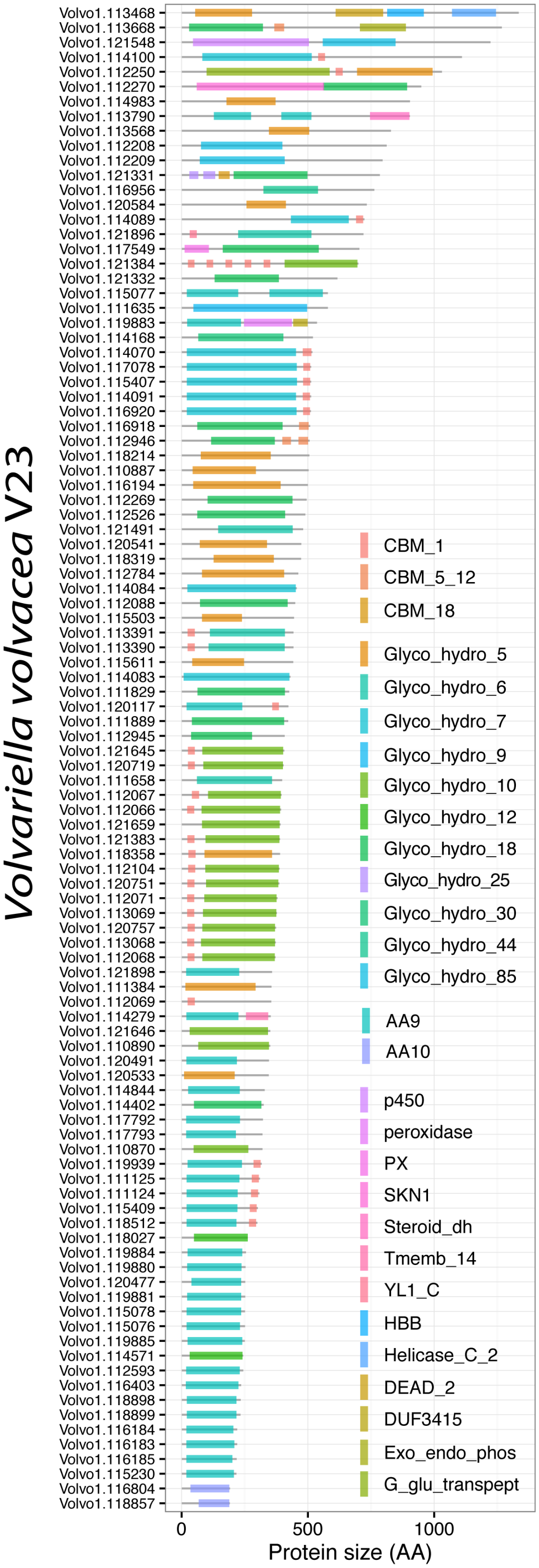

Figure S7. Identification of proteins involved in cellulose, xylan, and chitin deconstruction in *Chaetomium globosum* v1.0 (phylum Basidiomycota).

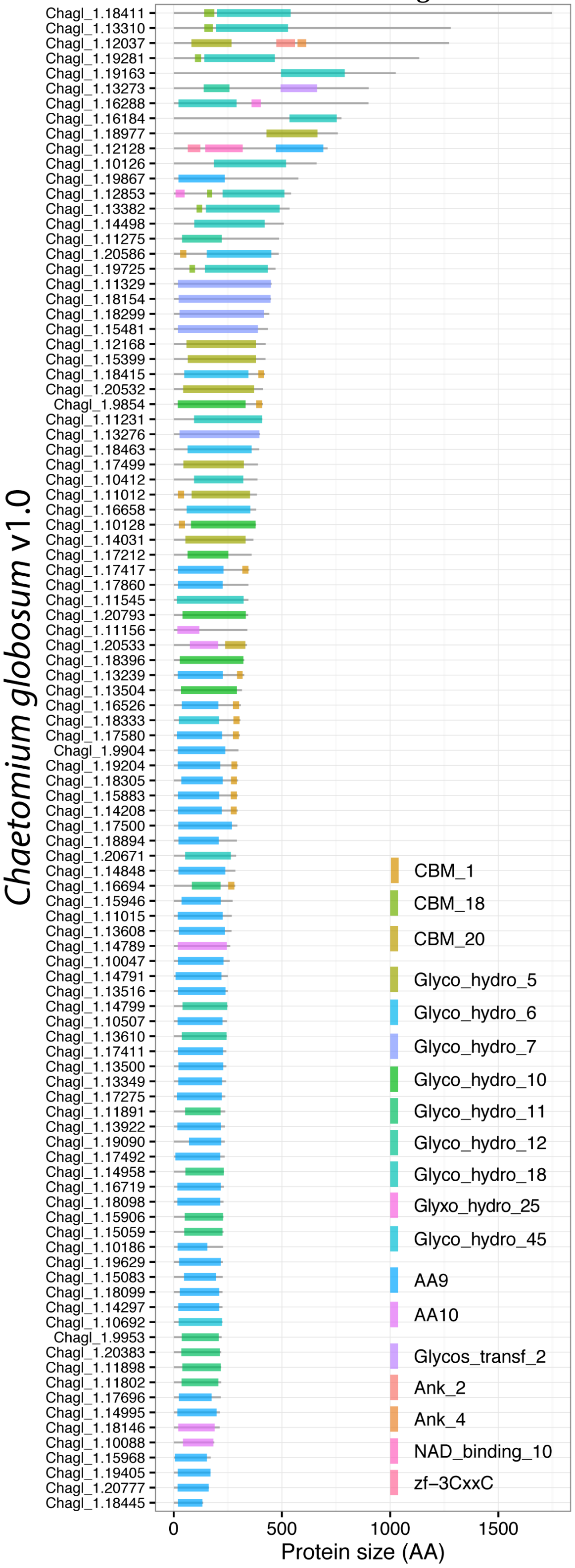

Figure S8. Identification of proteins involved in cellulose, xylan, and chitin deconstruction in *Aspergillus oryzae* RIB40 (phylum Ascomycota).

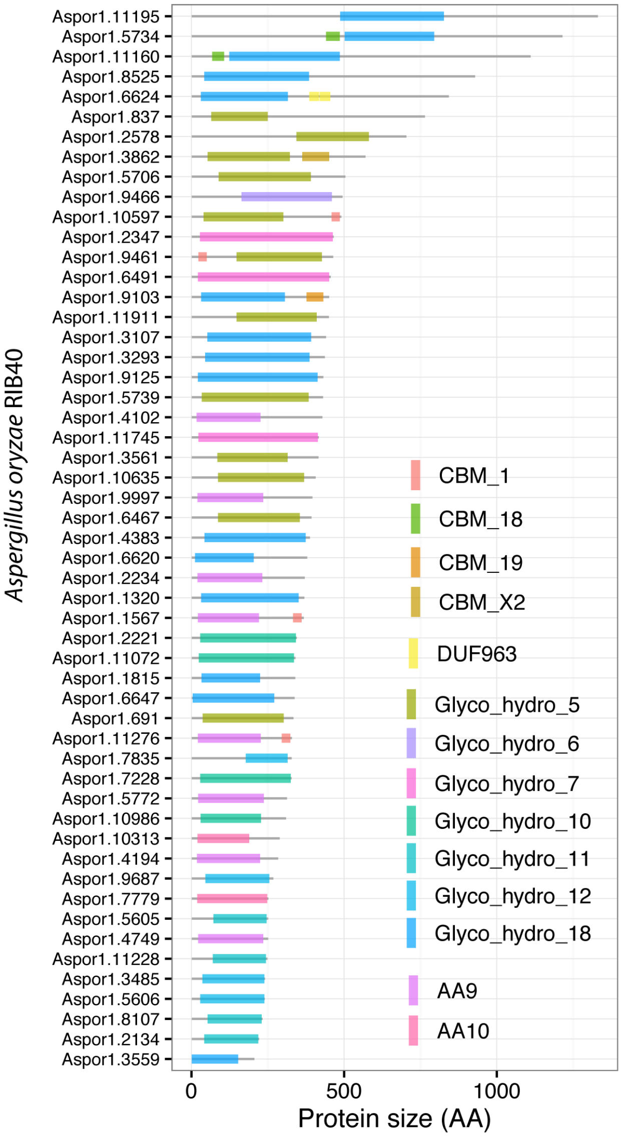

Figure S9. Identification of proteins involved in cellulose, xylan, and chitin deconstruction in *Postia placenta* MAD698-R v1.0 (phylum Basidiomycota).

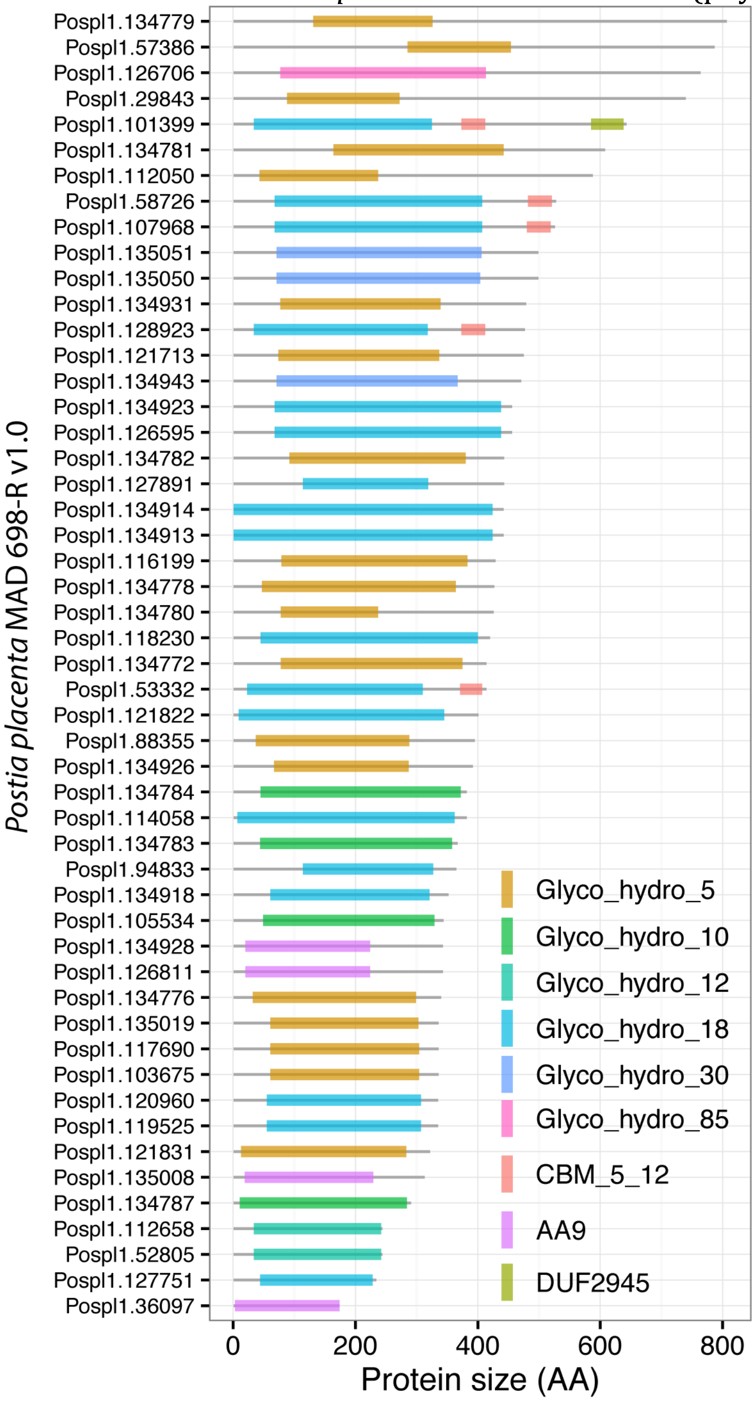

Figure S10. Identification of proteins involved in cellulose, xylan, and chitin deconstruction in *Trichoderma reesei* v2.0 (phylum Ascomycota).

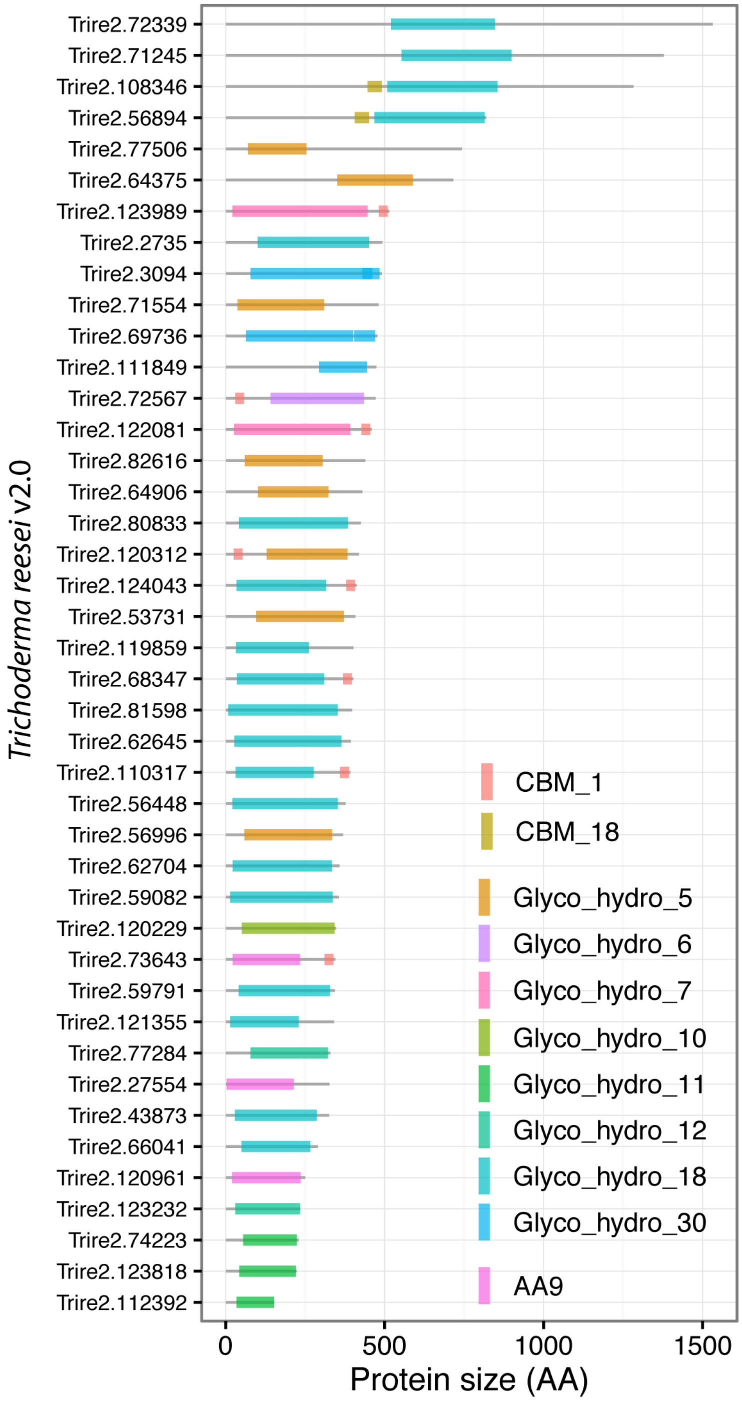

Figure S11. Identification of proteins involved in cellulose, xylan, and chitin deconstruction in *Myceliophthora thermophile* (*Sporotrichum thermophile*) v2.0 (phylum Ascomycota).

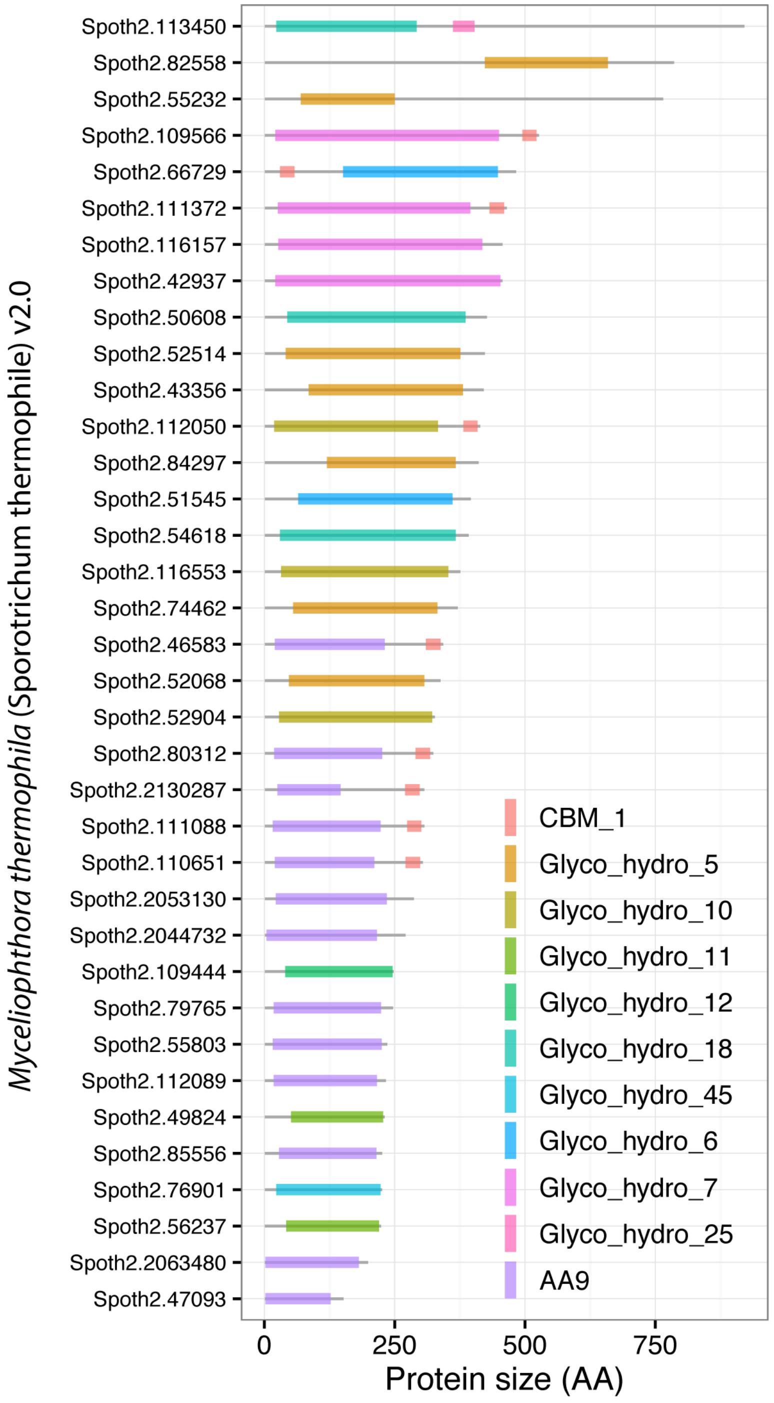

Table S1.

<table>
<tr><th>GH5
prot.</th><th>Multi-domain architecture (Multi-activity)</th><th colspan="2">Example (Strain – gene ID)</th></tr>
<tr><td>1</td><td>AAA-GH5</td><td>Monacrosporium haptotylum CBS 200.50</td><td>Monha1|8792</td></tr>
<tr><td>1</td><td>AA permease-GH5</td><td>Eutypa lata UCREL1</td><td>Eutla1|2714</td></tr>
<tr><td>3</td><td>3(CBM10) -GH5</td><td>Orpinomyces sp.</td><td>Orpsp1_1|1192248</td></tr>
<tr><td>2</td><td>2(CBM10)-GH5</td><td>Orpinomyces sp.</td><td>Orpsp1_1|1174941</td></tr>
<tr><td>1</td><td>CBM10-GH5</td><td>Orpinomyces sp.</td><td>Orpsp1_1|1175103</td></tr>
<tr><td>2</td><td>2(CBM1)-GH5</td><td>Fibulorhizoctonia sp. CBS 109695 v1.0</td><td>Fibsp1|1053206</td></tr>
<tr><td>192</td><td>CBM1-GH5</td><td>Monacrosporium haptotylum CBS 200.50</td><td>Monha1|7006</td></tr>
<tr><td>1</td><td>CBM1-GH5-2(AMP-binding)</td><td>Stereum hirsutum FP-91666 SS1</td><td>Stehi1|136401</td></tr>
<tr><td>8</td><td>CBM1-GH5-CBMX2</td><td>Verticillium dahliae v1.0</td><td>Verda1|8195</td></tr>
<tr><td>2</td><td>CBM1-2(GH5)</td><td>Sistotremastrum suecicum</td><td>Sissu1|1061495</td></tr>
<tr><td>2</td><td>CBM1-GH5-GH6</td><td>Sistotremastrum niveocremeum HHB9708 ss-1 1.0</td><td>Sisni1|485627</td></tr>
<tr><td>1912</td><td>GH5</td><td>Hydnomerulius pinastri</td><td>Hydpi2|44163</td></tr>
<tr><td>2</td><td>GH5-ALG3</td><td>Armillaria mellea</td><td>Armme1_1|9170</td></tr>
<tr><td>1</td><td>GH5-Ank 2-Ank 4</td><td>Chaetomium globosum v1.0</td><td>Chagl_1|12037</td></tr>
<tr><td>38</td><td>GH5-CBM1</td><td>Trichoderma virens Gv29-8 v2.0</td><td>TriviGv29_8_2|61403</td></tr>
<tr><td>2</td><td>GH5-CBM10</td><td>Orpinomyces sp.</td><td>Orpsp1_1|1183752</td></tr>
<tr><td>8</td><td>GH5-2(CBM10)</td><td>Orpinomyces sp.</td><td>Orpsp1_1|1180574</td></tr>
<tr><td>1</td><td>GH5-3(CBM10)</td><td>Orpinomyces sp.</td><td>Orpsp1_1|1188618</td></tr>
<tr><td>1</td><td>GH5-4(CBM10)</td><td>Orpinomyces sp.</td><td>Orpsp1_1|1188503</td></tr>
<tr><td>1</td><td>GH5-2(CBM1)</td><td>Peniophora sp. v1.0</td><td>Ricme1|754209</td></tr>
<tr><td>35</td><td>GH5-CBMX2</td><td>Neofusicoccum parvum UCRNP2</td><td>Neopa1|9276</td></tr>
<tr><td>4</td><td>2(GH5)</td><td>Pichia stipitis v2.0</td><td>Picst3|55209</td></tr>
<tr><td>2</td><td>2(GH5)-2(CBM10)</td><td>Orpinomyces sp.</td><td>Orpsp1_1|1185330</td></tr>
<tr><td>1</td><td>GH5-Choline kinase</td><td>Aspergillus terreus NIH 2624</td><td>Aspte1|4936</td></tr>
<tr><td>1</td><td>GH5-DEAD 2-HBB-Helicase C 2</td><td>Volvariella volvacea V23</td><td>Volvo1|113468</td></tr>
<tr><td>3</td><td>GH5-GH79C</td><td>Aureobasidium pullulans var. melanogenum CBS 110374</td><td>Aurpu_var_mel1|38401</td></tr>
<tr><td>2</td><td>GH5-HET</td><td>Zymoseptoria pseudotritici STIR04 2.2.1</td><td>Zymps1|798487</td></tr>
<tr><td>1</td><td>GH5-LOR</td><td>Metarhizium acridum CQMa 102</td><td>Metac1|2210</td></tr>
<tr><td>1</td><td>GH5-2(Myb DNA-bind 3)</td><td>Melampsora lini CH5</td><td>Melli1|199291</td></tr>
<tr><td>1</td><td>GH5-Peptidase A22B</td><td>Armillaria mellea</td><td>Armme1_1|117</td></tr>
<tr><td>1</td><td>GH5-RhoGEF</td><td>Malassezia sympodialis ATCC 42132</td><td>Malsy1_1|1737</td></tr>
<tr><td>3</td><td>GH5-Ricin B lectin</td><td>Arthrobotrys oligospora ATCC 24927</td><td>Artol1|4348</td></tr>
<tr><td>1</td><td>GH5-SKN1</td><td>Trametes versicolor</td><td>Trave1|62064</td></tr>
<tr><td>1</td><td>GH5-Xpo1</td><td>Alternaria brassicicola</td><td>AB01609.1-Alternaria_brassicicola</td></tr>
<tr><td>1</td><td>GH5-zf-Mss51</td><td>Hysterium pulicare</td><td>Hyspu1 1|118328</td></tr>
<tr><td>1</td><td>DSPc-GH5-UQ con</td><td>Rhizopus microsporus var. chinensis CCTCC M201021</td><td>Rhich1|4454</td></tr>
</table>

1	2(GTP EFTU D2)-IF-2-GTP EFTU D4-GH5	Rhytidhysteron rufulum	Rhyru1_1|116451
1	**G glu transpept-CBM1-GH5**	Volvariella volvacea V23	Volvo1|112250
1	Gln-synt C-GH5	Fibroporia radiculosa TFFH 294	Fibra1|2674
1	**Glyco tran 28 C-GH5**	Alternaria brassicicola	AB09769.1-Alternaria_brassicicola
1	**Lactamase B-GH5**	Diplodia seriata DS831	Dipse1|6209
1	2(Na Ca ex)-GH5-Cyclin	Phaeomoniella chlamydospora UCRPC4	Phach1|5684
2	PX-GH5	Dacryopinax primogenitus DJM 731 SSP1	Dacsp1|107572
1	Pirin-GH5	Diplodia seriata DS831	Dipse1|597
1	Proteasome-GH5	Wallemia ichthyophaga EXF-994	Walic1|176
3	Ricin B lectin-GH5	Cryptococcus neoformans var. grubii H99	Cryne_H99_1|706
1	**Sulfatase-MFS 1-GH5**	Zymoseptoria pseudotritici STIR04 2.2.1	Zymps1|795683
1	WSC-WSC-GH5	Tilletiaria anomala UBC 951	Tilan2|267361
GH6 # prot.	Multi-domain architecture (**Multi-activity**)	Example (Strain – gene ID)	
1	4(CBM10)-GH6	Orpinomyces sp.	Orpsp1_1|1182117
3	3(CBM10)-GH6	Orpinomyces sp.	Orpsp1_1|1174935
15	2(CBM10)-GH6	Orpinomyces sp.	Orpsp1_1|1180976
2	CBM10-GH6	Orpinomyces sp.	Orpsp1_1|1177562
2	**CBM1-GH5-GH6**	Sistotremastrum suecicum	Sissu1|1057361
86	CBM1-GH6	Stagonospora nodorum SN15	Stano2|12155
1	**GH3-Fn3-like-CBM10-GH6-2(CBM10)**	Orpinomyces sp.	Orpsp1_1|1176522
134	GH6	Stagonospora nodorum SN15	Stano2|9852
3	GH6-CBM1	Podospora anserina S mat+	Podan2|5491
1	**Peptidase M3-2(CBM10)-GH6**	Orpinomyces sp.	Orpsp1_1|1181647
1	YL1 C-GH6	Volvariella volvacea V23	Volvo1|121896
GH7 # prot.	Multi-domain architecture (**Multi-activity**)	Example (Strain – gene ID)	
1	Alpha-amylase-DUF1966-CBM20-GH7	Phaeomoniella chlamydospora UCRPC4	Phach1|4371
304	GH7	Rhytidhysteron rufulum	Rhyru1_1|111535
133	GH7-CBM1	Volvariella volvacea V23	Volvo1|116920
1	GH7-Pre-SET-SET	Colletotrichum fioriniae PJ7	Colfi1|283571
GH8 # prot.	Multi-domain architecture (**Multi-activity**)	Example (Strain – gene ID)	
12	GH8	Pseudozyma antarctica T-34	Psean1_1|80331
GH9 # prot.	Multi-domain architecture (**Multi-activity**)	Example (Strain – gene ID)	
1	BCNT-GH9	Rhizopus microsporus var. chinensis CCTCC M201021	Rhich1|1669
1	3(CBM10)-GH9	Orpinomyces sp.	Orpsp1_1|1183432
1	CBM10-GH9	Orpinomyces sp.	Orpsp1_1|1182044
2	CelD N-GH9	Aureobasidium pullulans var. pullulans EXF-150	Aurpu_var_pul1|367568
71	GH9	Bjerkandera adusta v1.0	Bjead1_1|150399

1	GH9-2(CBM10)	Orpinomyces sp.	Orpsp1_1|1177393
4	GH9-3(CBM10)	Orpinomyces sp.	Orpsp1_1|1183575
1	**Transposase 21-GH9**	Lichtheimia corymbifera JMRC:FSU:9682	Liccor1|6997
GH10 # prot.	Multi-domain architecture (**Multi-activity**)	Example (Strain – gene ID)	
1	Adaptin N-Alpha adaptinC2-GH10	Rhytidhysteron rufulum	Rhyru1_1|112915
1	2(CBM10)-GH10	Orpinomyces sp.	Orpsp1_1|1186194
3	CBM10-GH10	Orpinomyces sp.	Orpsp1_1|1176015
1	5(CBM1)-GH10	Volvariella volvacea V23	Volvo1|121384
107	CBM1-GH10	Pyrenophora teres f. teres	Pyrtt1|9468
2	**CBM1-2(GH10)**	Sphaerobolus stellatus	Sphst1|66307
1	**CBM1-3(GH10)**	Piriformospora indica DSM 11827 from MPI	Pirin1|75864
1	DUF676-GH10	Venturia pirina	Venpi1|209646
354	GH10	Omphalotus olearius	Ompol1|6647
51	GH10-CBM1	Neofusicoccum parvum UCRNP2	Neopa1|5657
2	GH10-2(CBM10)	Orpinomyces sp.	Orpsp1_1|1174840
1	GH10-3(CBM10)	Orpinomyces sp.	Orpsp1_1|1185862
1	**GH11-GH10-2(CBM10)**	Orpinomyces sp.	Orpsp1_1|1191833
1	**LIDHydrolase-GH10**	Ascocoryne sarcoides NRRL50072	Ascsa1|427
GH11 # prot.	Multi-domain architecture (**Multi-activity**)	Example (Strain – gene ID)	
1	CBM10-GH11	Orpinomyces sp.	Orpsp1_1|1190356
1	CBM1-GH11	Coprinopsis cinerea	Copci1|7658
306	GH11	Phaeoacremonium aleophilum UCRPA7	Phaal1|764
51	GH11-CBM1	Monacrosporium haptotylum CBS 200.50	Monha1|4361
3	GH11-2(CBM10)	Orpinomyces sp.	Orpsp1_1|1187914
2	**GH11-3(CBM10)-GH11**	Orpinomyces sp.	Orpsp1_1|1188064
1	**GH11-2(CBM10)-GH11**	Orpinomyces sp.	Orpsp1_1|1175252
1	**GH11-CBM10-GH11-CBM10-GH11**	Orpinomyces sp.	Orpsp1_1|1175428
1	**GH11-GH10-2(CBM10)**	Orpinomyces sp.	Orpsp1_1|1191833
4	**2(GH11)**	Orpinomyces sp.	Orpsp1_1|1191822
1	**GH11-Pkinase**	Hysterium pulicare	Hyspu1_1|120486
1	**GH11-Polysacc deac 1**	Orpinomyces sp.	Orpsp1_1|1181166
GH12 # prot.	Multi-domain architecture (**Multi-activity**)	Example (Strain – gene ID)	
3	CBM1-GH12	Colletotrichum higginsianum IMI 349063	Colhi1|3760
360	GH12	Bjerkandera adusta v1.0	Bjead1_1|173673
7	GH12-CBM1	Galerina marginata	Galma1|61235
1	**GH12-3(Cu-oxidase)**	Puccinia graminis f. sp. tritici	Pucgr2|14446
2	**GH12-Glycos transf 2**	Chaetomium globosum v1.0	Chagl_1|13273
2	Rieske-Ring hydroxyl A-GH12	Aspergillus fumigatus Af293 from AspGD	Aspfu1|3383
1	SKG6-GH12	Diplodia seriata DS831	Dipse1|8169

GH18 # prot.	Multi-domain architecture (**Multi-activity**)	Example (Strain – gene ID)	
1	AAA-GH18	Glarea lozoyensis ATCC 20868	Glalo1|12292
1	**Amidase-GH18**	Rhodosporidium toruloides IFO0880 v2.0	Rhoto_IFO0880_2|3656
1	3(Ank 2)-GH18	Hysterium pulicare	Hyspu1_1|119341
1	3(CBM18)-GH18	Nectria haematococca v2.0	Necha2|45570
6	2(CBM18)-GH18	Fusarium fujikuroi IMI 58289	Fusfu1|2308
205	CBM18-GH18	Trichoderma virens Gv29-8 v2.0	TriviGv29_8_2|112097
1	CBM18-GH18-Ank 2	Sclerotinia sclerotiorum v1.0	Sclsc1|773
1	CBM18-GH18-CBM18	Glarea lozoyensis ATCC 20868	Glalo1|7011
1	CBM18-GH18-2(CBM18)	Magnaporthe grisea v1.0	Maggr1|119428
2	CBM18-GH18-MU117	Aspergillus niger ATCC 1015	Aspni7|1160118
1	**CBM18-GH18-Peptidase S8**	Glarea lozoyensis ATCC 20868	Glalo1|6457
1	**FAD binding 4-FAD-oxidase C-GH18**	Eutypa lata UCREL1	Eutla1|2587
1	GFA-GH18	Cladosporium fulvum	Clafu1|188744
1704	GH18	Melampsora lini CH5	Melli1|206913
1	GH18-Ank 2-Ank 4	Diplodia seriata DS831	Dipse1|3459
41	GH18-CBM1	Cordyceps militaris CM01	Cormi1|5672
15	GH18-CBM19	Kluyveromyces lactis	Klula1|1325
136	GH18-CBM5/12	Coniophora puteana	Conpu1|114748
6	GH18-2(CBM5/12)	Dichomitus squalens	Dicsq1|75136
1	GH18-CBM5/12-DUF2945	Postia placenta MAD 698-R v1.0	Pospl1|101399
2	GH18-CBM5/12-Exo endo phos	Plicaturopsis crispa	Plicr1|179671
3	GH18-CBM18	Ascocoryne sarcoides NRRL50072	Ascsa1|10128
1	GH18-2(CBM18)	Colletotrichum graminicola M1.001	Colgr1|3876
2	GH18-2(DUF963)	Aspergillus oryzae RIB40	Aspor1|6624
1	GH18-Erf4	Phycomyces blakesleeanus NRRL1555 v2.0	Phybl2|180075
1	GH18-Erf4-2(ATP-synt C)	Rhizopus microsporus var. chinensis CCTCC M201021	Rhich1|9837
1	GH18-Flocculin t3	Pichia stipitis v2.0	Picst3|48142
1	GH18-2(Flocculin t3)	Debaryomyces hansenii	Debha1|2163
1	**GH18-Fungal trans 2**	Oidiodendron maius Zn v1.0	Oidma1|178807
6	**2(GH18)**	Coniophora puteana	Conpu1|101782
1	**GH18-GH81**	Macrophomina phaseolina MS6	Macph1|905
1	GH18-HET	Marssonina brunnea f. sp. multigermtubi MB m1	Marbr1|4927
30	GH18-Hce2	Aspergillus kawachii IFO 4308	Aspka1_1|13306
5	GH18-GH25	Myceliophthora thermophila (Sporotrichum thermophile) v2.0	Spoth2|113450
1	GH18-MFS 1	Aspergillus clavatus NRRL 1 from AspGD	Aspcl1|5506
1	GH18-Methyltransf 16	Monacrosporium haptotylum CBS 200.50	Monha1|2239
3	GH18-PhoD	Uncinocarpus reesii 1704	Uncre1|3702
1	GH18-Ricin B lectin	Phlebia brevispora HHB-7030 SS6	Phlbr1|150005

1	GH18-Sld5	Rhizopus oryzae 99-880 from Broad	Rhior3|10669
13	GH18-UBA	Mycosphaerella graminicola v2.0	Mycgr3|71099
2	GH18-Vps53 N	Diplodia seriata DS831	Dipse1|5751
1	GH18-WSC	Phialocephala scopiformis 5WS22E1 v1.0	Phisc1|142355
1	IDO-GH18	Setosphaeria turcica Et28A	Settu1|25278
1	Inhibitor I9-GH18	Rhodosporidium toruloides IFO0559 1	Rhoto_IFO0559_1|3780
9	**GH25-CBM18-GH18**	Exidia glandulosa	Exigl1|829073
18	**GH25-GH18**	Aspergillus niger ATCC 1015	Aspni7|1177639
4	**GH25-GH18-Hce2**	Grosmannia clavigera kw1407	Grocl1|7933
2	**2(GH25)-CBM18-GH18**	Microdochium bolleyi J235TASD1 v1.0	Micbo1|220466
1	**2(GH25)-GH18**	Eutypa lata UCREL1	Eutla1|3713
1	**8(GH25)-GH18**	Allomyces macrogynus ATCC 38327	Allma1|9612
1	PIF1-PX-GH18	Armillaria mellea	Armme1 1|11040
30	PX-GH18	Daedalea quercina	Daequ1|708742
1	**Pectate lyase 3-GH18**	Aureobasidium pullulans var. namibiae CBS 147.97	Aurpu_var_nam1|80126
1	**Pkinase-GH18**	Setosphaeria turcica Et28A	Settu1|47026
5	**Pkinase-GH18-UBA**	Cochliobolus miyabeanus ATCC 44560	Cocmi1|92644
1	RTA1-GH18	Fusarium oxysporum f. sp. lycopersici 4287 v2	Fusox2|25975
1	SKN1-GH18	Volvariella volvacea V23	Volvo1|112270
1	Sas10-CBM18-GH18	Botrytis cinerea v1.0	Botci1|5655
GH30 # prot.	Multi-domain architecture (**Multi-activity**)	Example (Strain – gene ID)	
1	Adaptin N-GH30	Omphalotus olearius	Ompol1|2830
110	GH30	Hypholoma sublateritium	Hypsu1|199806
36	**2(GH30)**	Trichoderma reesei v2.0	Trire2|69736
14	GH30	Stagonospora nodorum SN15	Stano2|1204
1	GH30-CBM1	Aspergillus clavatus NRRL 1 from AspGD	Aspcl1|1077
GH44 # prot.	Multi-domain architecture (**Multi-activity**)	Example (Strain – gene ID)	
22	GH44	Coprinopsis cinerea	Copci1|11421
2	GH44-CBM1	Fibulorhizoctonia sp. CBS 109695 v1.0	Fibsp1|378277
2	**GH44-Glyco transf 22**	Agaricus bisporus var. burnettii JB137-S8	Agabi_varbur_1|33905
GH45 # prot.	Multi-domain architecture (**Multi-activity**)	Example (Strain – gene ID)	
1	3(CBM10)-GH45	Orpinomyces sp.	Orpsp1_1|1186105
2	2(CBM10)-CBM1-GH45	Orpinomyces sp.	Orpsp1_1|1180594
3	2(CBM10)-GH45	Orpinomyces sp.	Orpsp1_1|1174556
1	CBM10-CBM1-GH45	Orpinomyces sp.	Orpsp1_1|1181902
1	5(CBM1)-GH45	Pichia pastoris	Picpa1|40019
1	2(CBM1)-GH45	Lichtheimia corymbifera JMRC:FSU:9682	Liccor1|11476
9	CBM1-GH45	Rhizopus oryzae 99-880 from Broad	Rhior3|6450

105	GH45	Leptosphaeria maculans	Lepmu1|3443
23	GH45-CBM1	Nectria haematococca v2.0	Necha2|94623
1	GH45-2(CBM1)	Pyrenophora tritici-repentis	PTRG_05235
1	GH45-3(CBM1)	Pyrenophora teres f. teres	Pyrtt1|344
GH48 # prot.	Multi-domain architecture (**Multi-activity**)	Example (Strain – gene ID)	
6	GH48	Orpinomyces sp.	Orpsp1_1|1183188
4	GH48-2(CBM10)	Orpinomyces sp.	Orpsp1_1|1181284
1	GH48-3(CBM10)	Orpinomyces sp.	Orpsp1_1|1191449
GH85 # prot.	Multi-domain architecture (**Multi-activity**)	Example (Strain – gene ID)	
1	DASH Dam1-GH85	Arthrobotrys oligospora ATCC 24927	Artol1|3267
93	GH85	Laetiporus sulphureus var. sulphureus	Laesu1|639294
1	SRP-alpha N-SRP54 N-SRP54-GH85	Pyronema confluens CBS100304	Pyrco1|5044
1	p450-GH85	Volvariella volvacea V23	Volvo1|121548
AA9 # prot.	Multi-domain architecture (**Multi-activity**)	Example (Strain – gene ID)	
1	Aldedh-AA9-Gly-zipper Omp	Omphalotus olearius	Ompol1|7713
1	CDC24-RhoGEF-PH 10-PB1-AA9-Aminotran 1 2	Phaeomoniella chlamydospora UCRPC4	Phach1|5619
1	Cpn10-AA9	Metarhizium robertsii ARSEF 23	Metan1|6336
1425	AA9	Phialocephala scopiformis 5WS22E1 v1.0	Phisc1|781244
1	AA9-ASF1 hist chap	Monacrosporium haptotylum CBS 200.50	Monha1|1425
1	AA9-Ank 4	Omphalotus olearius	Ompol1|64
335	AA9-CBM1	Cochliobolus victoriae FI3	Cocvi1|89820
5	AA9-2(CBM1)	Sphaerobolus stellatus	Sphst1|778601
1	AA9-6(CBM1)	Monacrosporium haptotylum CBS 200.50	Monha1|9663
1	AA9-28(CBM1)	Arthrobotrys oligospora ATCC 24927	Artol1|5689
3	AA9-CFEM	Melampsora lini CH5	Melli1|203374
1	AA9-Cyt-b5	Colletotrichum fioriniae PJ7	Colfi1|277833
1	AA9-DUF1388	Aspergillus clavatus NRRL 1 from AspGD	Aspcl1|1225
1	**AA9-Fungal trans**	Colletotrichum fioriniae PJ7	Colfi1|281053
1	**AA9-GH114**	Hysterium pulicare	Hyspu1_1|113907
7	**AA9-AA9**	Omphalotus olearius	Ompol1|4884
**1**	**AA9-AA9-Steroid dh**	Volvariella volvacea V23	Volvo1|113790
1	AA9-Tmemb 14	Volvariella volvacea V23	Volvo1|114279
1	**AA9-peroxidase-DUF3415**	Volvariella volvacea V23	Volvo1|119883
1	MFS 1-AA9	Glarea lozoyensis ATCC 20868	Glalo1|9471
1	MR MLE N-MR MLE C-PWI-AA9	Armillaria mellea	Armme1_1|1766
1	Mod r-AA9-CBM1	Armillaria mellea	Armme1_1|5851
1	Mod r-AA9-CBM1-NCA2-2(HEAT 2)	Omphalotus olearius	Ompol1|394
1	Prefoldin-AA9	Wallemia ichthyophaga EXF-994	Walic1|1190
1	zf-3CxxC-NAD binding 10-AA9	Chaetomium globosum v1.0	Chagl_1|12128

AA10<br># prot.	Multi-domain architecture (**Multi-activity**)	Example (Strain – gene ID)	
1	DnaJ-DnaJ-X-AA10	Armillaria mellea	Armme1_1|13623
135	AA10	Hypholoma sublateritium	Hypsu1|199738
3	AA10-CBM1	Venturia pirina	Venpi1|214688
31	AA10-CBM20	Cochliobolus heterostrophus C5 v2.0	CocheC5 3|1096518
1	AA10-Ribosomal L12 N-Ribosomal L12	Phaeomoniella chlamydospora UCRPC4	Phach1|188
